# T Cell/Mast Cell Crosstalk in the Skin of Patients Suffering From Immune‐Mediated Diseases, Focusing on Chronic Spontaneous Urticaria

**DOI:** 10.1002/clt2.70145

**Published:** 2025-12-29

**Authors:** Elias Toubi, Raeda Mubariki, Zahava Vadasz

**Affiliations:** ^1^ Allergy Service Holy Family Hospital Nazareth Israel; ^2^ Rappaport Faculty of Medicine Technion Haifa Israel; ^3^ Bnai Zion Medical Center Haifa Israel

**Keywords:** cell proximity, crosstalk, CSU, polarization

## Abstract

The crosstalk between immune cells in skin lesions of numerous immune‐mediated diseases such as atopic dermatitis, psoriasis and other T cell mediated is fundamental for understanding the pathogenesis and treating these diseases. The mechanisms by which most activated immune cells such as T cells, mast cells, and neutrophils are shifted from peripheral blood into inflamed skin, and by which they interact, are complex and different in all these diseases. However, once immune cells are infiltrated into the skin, they are polarized to be in close proximity to each other, leading to their further activation and the production of pro‐inflammatory cytokines. The immune synapse in patients' skin suffering from chronic spontaneous urticaria (CSU) is fundamental for mast cell activation and degranulation. The role of T cell‐mast cell proximity in the pathogenesis of CSU is fairly assessed. A better understanding of this scenario is important for developing beneficial therapies when standard treatment fails to achieve remission.

**Abbreviations**
Btk: Bruton tyrosine kinase
CSU: Chronic spontaneous urticarial
FcεRI: High‐affinity IgE receptor
LPS: Lipopolysaccharides
MCs: Mast cells
MRGPRX2: Mas‐Related G‐Protein Coupled Receptor‐X2
TF: Tissue factor
TLRs: Toll like receptors
TNF‐α: Tumor necrosis factor alpha
Tregs: T regulatory cells

## 1 Introduction

The polarization of, and the crosstalk between immune cells in inflamed tissue, have been discussed for several decades and are fundamental in the pathogenesis of many allergic diseases, namely, in the lesional skin of these diseases. The character and mechanisms of this crosstalk is different depending on the disease group, thus involving different immune cells. Mast cells (MCs) are efficient antigen‐presenting cells, mainly for T cells, resulting in their activation and proliferation, by MHC class I and II‐dependent mechanisms. This was shown to be IFN‐γ dependent, which induces HLA class II, CD80, and CD40 expression on mast cells. IFN‐ γ‐primed *MCs* guide activation of T cells by *staphylococcus aureus* superantigen. Following this, *MCs* also release many chemotactic molecules and pro‐inflammatory cytokines such as TNF‐α, MMPs, and IL‐6, inducing T‐cell migration into inflamed tissues [1, 2]. In addition, *MCs* are activated via cell surface receptors, such as FcεRI, toll‐like receptors (TLR) and cytokine receptors, contributing by this to allergic reactions and tissue inflammation. However, excessive *MC* activation was also shown to induce regulatory functions by their crosstalk with T cells [3]. Effector CD4+ T cells are major residents in sites of tissue inflammation strategically located near blood vessels, thus inducing the activation and degranulation of tissue MCs and basophils. Activated CD4+ T cells induce specific transcriptomic program in MCs, namely, the production of cytokines, chemokines, prostaglandins and other pro‐inflammatory cytokines, contributing to a crosstalk process between all immune cells. Immune responses such as the release of Th1 and Th2‐ related cytokines, as well as the expression of adhesion molecules on endothelial cells are crucial in modulating the immune synapse and the close proximity between mast cells, dendritic cells, and T cells in modulating adaptive immune responses [4]. In this review, we aim to summarize the status of the synapse and crosstalk between activated T cells and MCs, focusing on the role of this crosstalk in the pathogenesis of chronic spontaneous urticaria (CSU).

## 2 The Role of MCs in Inducing Immune Cell Polarization

**FIGURE 1:**
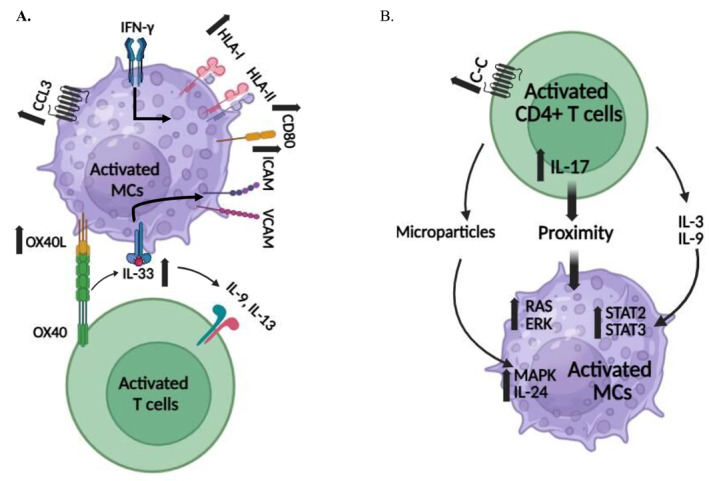
(A) Activated MCs increase CD4+ T cell activity and their cell to cell proximity by: 1‐ IFN‐γ stimulation and increased Ag‐presenting capacity. 2‐ OX40L‐OX40 binding increases IL‐33 expression. 3‐ IL‐33 mediated IL‐9 and IL‐13 secretion and increased expression of adhesive molecules. (B) Activated CD4+ T cells increase MC's proximity by increased C‐C expression, IL‐17 expression and IL‐3. IL‐9 secretion.

Mast cells have long been considered the main effector cells in the pathogenesis of allergic diseases. As a source of a broad spectrum of pro‐inflammatory cytokines and chemokines, mast cells have a role in inducing both innate and adaptive immune responses, namely, the activation of both dendritic cells (DCs) and T cells. In this respect, activated MCs are fundamental in various T cell‐mediated inflammatory processes turning out to be in close proximity to T cells [5]. *The cell‐to‐cell interaction between MCs and T cells* *: MCs* may interact with T cells in a cell‐to‐cell interaction via membrane‐associated receptors, such as FcεRI and TLRs, the release of pro‐inflammatory cytokines, such as IL‐6, IL‐17 and chemokines [6, 7]. Growing evidence indicates that microparticles play a pivotal role in cell‐to‐cell communication between MCs and T cells. Emerging evidence points to the ability of mast cells to be a rich source of secreted exosomes and microparticles, contributing to the activation and polarization of nearby cells. These particles are released from the endosomal compartment, or fragments shed from MC membranes as microparticles and are perceived to function as small stimulatory cells [8, 9]. *The role of MC chemokines in recruiting T cells:* In one study, resting tonsillar mast cells were shown to express high levels of chemokines such as CCL3. They were capable of recruiting T cells into close proximity to these mast cells. In addition, by expressing increased OX40 ligand (a TNF‐receptor superfamily), tonsillar mast cells induced T‐cell proliferation, especially following the aggregation of FcεRI on these mast cells. This study suggests that human MCs are very important in inducing adaptive immunity, by increasing their antigen‐presenting capacity to CD4+ T cells, thereby enhancing the close proximity between MCs and T cells [10]. *Mast cells are antigen presenting cells interacting with T cells by expressing adhesion molecules:* Stem cell‐derived mast cells (CD34+ cells), were shown to act as antigen presenting cells, increasing their presenting capacity following their stimulation with IFN‐γ. This increased their expression of HLA‐DR and CD80 leading to their enhanced proximity and crosstalk with CD4+ T cells [11]. In addition to being antigen‐presenting cells, MCs interact with DCs, T cells, and B cells by expressing adhesion molecules and releasing various cytokines and chemokines. They mediate their immune‐inflammatory responses through many cytokines such as TNF‐α. TNF‐α rapidly induces MC expression of many adhesive proteins, including intracellular adhesion molecule‐1 (ICAM‐1), vascular cell adhesion molecule‐1 (VCAM‐1), integrins, and L‐selectin, enabling them to interact closely and create structures such as focal adhesion sites, as well as triggering signaling pathways [12]. *The role of IL‐33 in mast/T cell proximity:* IL‐33 is an alarmin cytokine (alarm signal), with crucial roles in tissue homeostasis, allergic and non‐allergic inflammation. It targets group 2 innate lymphoid cells (ILCs), MCs, T regulatory cells (Tregs), Th1 and Th2 cells [13]. IL‐33 activates and re‐shapes MCs enhancing by that the production of CCL1, IL‐5, IL‐18 and IL‐13, by inducing phosphorylation of P38 and NF‐kB activation. This instructs skin MCs to efficiently co‐operate with other immune cells amplifying their crosstalk and interaction [14]. In one line with the above, a recent study showed that IL‐33‐primed human MCs were able to influence Th cell cytokine production, by promoting IL‐9 and increased IL‐13 production in Th cells via an OX40L‐dependent mechanism [15]. When activated by anti‐IgE, mast cells release growth factors and other mediators such as substance P. The latter induces VEGF and IL‐33, both of which contribute to increased angiogenesis and inflammation, which links mast cells and VEGF to the process of endothelial cell activation [16]. In a recent study, enhancement of histaminergic itch by IL‐33 was shown to rely on a mast cell‐ and IL‐33 dependent mechanism. By releasing histamine, tryptase, IL‐1, and TNF‐α, MCs were shown to play a role in central nervous system inflammatory disorders, through their interaction with neuropeptides that mediate endothelial cell activation [17, 18] (Figure 1A).

## 3 Activated T Cells Are Fundamental in Inducing MC Activation in Inflamed Tissues

Several decades ago, activated T cells were shown to activate MCs and induce their degranulation in inflamed tissues. When MCs were co‐cultured with activated T cells, they released pro‐inflammatory mediators such as histamine and TNF‐α. This process was prevented by separating them with a porous membrane, indicating that the contact between T cells and mast cells is responsible for mast cell activation and the production of pro‐inflammatory cytokines [19]. *Downstream signaling molecules in activated T cells:* The activation of human MC signaling pathways was shown to develop following their contact with activated T cells, resulting in downstream events, namely, the activation of Ras, NFkB and the release of pro‐inflammatory cytokines, such as IL‐6, IL‐4 and IL‐17. Additionally, the contact between human MCs and activated CD4+ T cells resulted in sustained ERK activation, which is associated with an increased dwell time at the nucleus as well as an increased IL‐8 release. Thus, ERK activation in human *MCs* is considered one of the mechanisms through which mast cells are activated and generate different mediators upon their close contact with T cells [20]. *T cell related cytokines and MC activation:* Among the many notable stimulatory cytokines, causing mast cell growth, migration, and inflammatory responses is IL‐3. It is also termed as a multi‐colony‐stimulating factor, predominantly produced by activated T cells. By interacting with MCs, IL‐3 has a role in enhancing IgE‐mediated reactions, as well as the development of tissue inflammatory responses [21]. MCs were also shown to be activated by Th9 and the production of IL‐9 by these cells. IL‐9 influences mast cells by activating STAT1, STAT2, and STAT3, in the presence of IL‐4 and transforming growth factor beta (TGF‐β). Downstream of IL‐4, other transcription factors such as STAT6, interferon regulatory 4 (IRF4), and nuclear factor of activated T cells (NFAT) are also involved in MC activation [22]. The close opposition of MCs and CD8+ T cells has also been reported in the skin of several immune‐mediated disorders. This close contact induced the up regulation of MC costimulatory molecules and the release of inflammatory products such as CCL5 and TNF‐α [23]. *The release of microparticles by activated T cells:* A fundamental mechanism by which activated T cells induce MC activation is the release of microparticles from these T cells when they are in close proximity to MCs. In an elegant study, microparticles were isolated from supernatants of activated T cells by high‐speed centrifugation and were characterized by the expression of integrin LFA‐1. Microparticles induced MC degranulation and IL‐8 release involving MARK signaling pathway. Mast cell activation and degranulation were also induced when stimulated by activated fixed T cells or by whole membranes of these T cells [24]. In this respect, uptake and internalization of activated T cell‐derived microparticles into human mast cells occurred within 24 h. Microparticles induced the up regulation of IL‐24, which is considered a hallmark of microparticles‐induced activation [25]. *T regulatory cells and MCs:* The interaction between CD4+CD25+ Treg cells with mast cells was assessed to show how this interaction possibly suppresses mast cell activation and degranulation. Indeed, Tregs inhibited mast cell degranulation via cell‐to‐cell contact, through OX40‐OX40 ligand crosstalk between Tregs and mast cells. The suppressive effect of Tregs was associated with increased cAMP concentration and reduced Ca (2+) influx. When T regs were depleted, IgE‐mediated mast cell, responses were restored [26]. *The role of γδ T cells in immune responses*: The presence of this subset of T cells, mainly in mucosal and epithelial tissues is considered to bridge the innate and adaptive immunity. Newly recruited and locally proliferating γδ T cells were the first T cell subset to respond to MC‐driven inflammation followed by increased IFN‐ϫ secretion. In many studies, MC‐ γδ T crosstalk was reported to be present in inflammatory peripheral tissues, thereby suggesting that this immune synapse is a fundamental mechanism in immunity at inflammation sites [27]. The issue of T cell/mast cell proximity in driving inflammatory immune responses remains a fundamental mechanism whose understanding is the subject of many ongoing research projects. However, the role of this important process in the pathogenesis of CSU is rarely reported (Figure 1B).

## 4 The Crosstalk Between Immune Cells in the Pathogenesis of CSU

In most cases, CSU is characterized by the spontaneous development of itchy wheals and angioedema and is regarded as autoimmune in origin. The autoimmune basis is evidenced by autologous serum skin test, basophil activation test, and the presence of IgG autoantibodies against IgE or its high‐affinity receptor (FcεRI) on MCs. In CSU, more than 50% of patients were found to have contemporary IgE and IgG autoantibodies, at least to one of the following autoantigens: IgE receptors, tissue factor, and thyroglobulins. Twenty‐five percent of patients had levels of anti‐FcεRI IgE, suggesting that it could be a novel autoantigen in CSU [28, 29]. Blood CD4+ T cells proliferation to FcεRI on MCs was detected in 27% of CSU patients, compared to 0% of controls. IFN‐ϫ responses to FcεRI was reported in 53% of subjects with CSU, and were detected earlier than autoantibodies against FcεRI. These results indicate the presence of antigen/disease‐specific autoreactive T cells in CSU [30]. The crosstalk between innate immune cells (neutrophils, MCs, eosinophils, and basophils) and adaptive immune cells (T cells and B cells) in the pathogenesis of CSU is complex. It includes soluble inflammatory factors, adhesion molecules, chemokines, microparticles, and cell‐to‐cell contacts. This takes place in both the peripheral blood and the skin of CSU patients. *In peripheral blood*: MCs are rarely found in peripheral blood of healthy individuals. Lin‐CD117+CD34+FceRI+ progenitor MCs were found to be increased in peripheral blood of CSU patients. Increased CD117+CD34+FcεRI‐chymase‐MC populations were also found increased in non‐healthy other than CSU individuals. Aiming to define the role of peripheral blood‐derived human MCs in CSU, they were activated by anti‐FcεRI antibodies. In this case, they produce histamine, TNF‐α, IL‐4, and IL‐6, contributing to T‐cell activation and to their recruitment into the skin of CSU patients [31, 32, 33]. *Peripheral serum markers in CSU:* Over many years, many serum inflammatory cytokines were investigated as possible biomarkers for CSU disease severity. Both Th1 and Th2 related pro‐inflammatory cytokines were found to be increased in the serum of CSU patients. Plasma IL‐17 and IL‐33 concentrations were significantly higher in CSU patients when compared with healthy individuals. Severe CSU patients had significantly higher IL‐17 and IL‐33 than moderate and mild ones. In a later study, serum levels of IL‐4, IL‐31, and IL‐17, showed the strongest correlation with the severity of CSU, suggesting their involvement in exacerbating the disease condition [34, 35]. Th17 cells and related cytokines (IL‐17 and IL‐21) were found to be significantly increased, and positively correlated with CSU disease severity. On the other hand, Treg‐related cytokines (TGF‐β and IL‐35) were significantly decreased; and negatively associated with CSU severity. The above involvement of T‐related cytokines in the pathogenesis of CSU encouraged the usage of cyclosporine A, which for over a decade, remained the only beneficial therapy until omalizumab was approved [36, 37]. The up regulation of coagulation factors, D‐dimers, and MMP9, were also reported to be biomarkers for disease severity in sera of CSU patients. In another study, the effect of increased levels of LPS, TNF‐α, IL‐33, VEGF, and histamine, on the expression of tissue factor (TF) on vascular endothelial cells was assessed. The expression of TF on endothelial cells was significantly increased following its co‐stimulation with the above CSU‐related molecules [38, 39]. Increased serum levels of the alarmin triad‐IL25‐IL‐33 and TSLP was found to be in significant correlation with UAS7 and was suggested to be a prognostic biomarker in CSU [40]. Serum miR‐125a‐5p and CCL‐17 are up regulated in CSU, and are correlated with treatment response, suggesting that they serve as potential serum biomarkers for CSU. In a very recent study, serum Mas‐Related G‐Protein Coupled Receptor‐X2 (MRGPRX2) and chemokine (C‐C motif) ligand 2 were found to be increased in the sera of CSU and their increase was in correlation with disease activity [41, 42]. *In the skin of CSU patients:* The core link in the pathogenesis of CSU is the activation of MCs, T cells, eosinophils and other immune cells infiltration around the small venules of the lesion. Increased vascular permeability, and recruitment of inflammatory cells directly dependent on MC mediators' release [43]. *Cytokines\chemokines and CD4+ T cells in the skin of CSU patients:* In cutaneous biopsies taken from patients with urticaria, multiple pro‐inflammatory cytokine and chemokine genes (IL‐6, CXCL8) were up regulated. This was associated with rapid mast cell STAT3 activation and the recruitment of inflammatory immune cells [44]. Over many decades, the excessive infiltration of CD4+ T cells in the skin of a selection of CSU patients considered this disorder to be a T cell‐mediated disease. Increased expression of Th2‐related cytokines (IL‐4 and IL‐5) in skin biopsies of CSU patients suggested their role in MC degranulation, and wealing. Considering this, the role of IL‐33, IL‐25, and thymic stromal lymphopoietin (TSLP) in the pathogenesis of CSU was assessed. Significant elevations of IL‐33+, IL‐25+, and TSLP+ cells were found in the dermis of lesional CSU skin and observed to be localized to endothelial cells and tryptase+ MCs, strongly supporting the involvement of Th2 cells in the pathogenesis of CSU [45]. In inflamed skin, IL‐33 is expressed on different types of cells, such as epithelial cells and endothelial cells. Following immune mediated signals, different types of cells such as basophiles, MCs and T cells recognize released IL‐33. The binding of IL‐33 to these cells, requires the expression of the specific receptor ST2, leading to the production of pro‐inflammatory cytokines and chemokines, and the close interaction between infiltrating cells in inflamed skin tissues [46]. *T cell\mast cell interaction in lesional CSU skin:* The classical physical contact between activated T cells and MCs is mostly present at the site of inflamed tissues. Mast cell‐released chemokines such as CCL5 have a role in recruiting CD8+ T cells to inflammatory sites. They can also present antigens by classes MHC II and I to CD4+ T cells. Immune infiltration analyzes indicated that T cells, *MCs*, and macrophages were increased in lesional CSU skin. By releasing mediators such as histamine, TNF‐α, MMP‐9, IL‐6, and others, *MCs* contribute to T‐cell activation, recruitment, and extravasation into inflamed tissues, suggesting this process to be fundamental in inducing adaptive immune responses. The release of these cytokines by *MCs* and their contribution to T‐cell activation is essential for leukocyte extravasation and T‐cell‐mediated inflammatory processes [47, 48, 49]. As mentioned above, activated CD4+ T cells are capable of directly inducing MC activation, by releasing microparticles both by their close contact or by their close interaction. MCs and activated T cells interact via ICAM‐1 and LFA‐1 expressed on their surface, and this interaction enhances FcεRI‐mediated MC degranulation and histamine release [50]. Currently, in addition to the Th1 and Th2 immune response in the skin of CSU patients, the role of Th17 is becoming increasingly important. All together, these different T cell subsets, promote in a kind of orchestra, the crosstalk between them and MCs. One of the additional mechanisms of this interaction is the increased expression of histamine receptor HR4 on T cells followed by the activation of Th2 and Th17 cells. Even though it is increased in the lesional skin of CSU patients, the possible role of their proximity in the pathogenesis of CSU was not confirmed [51, 52]. In a previous study, the increased expression of CD4+ T cells and MCs was reported in both lesional and non‐lesional skin of CSU patients. Both types of cells expressed elevated levels of IL‐17 and were found in close proximity to each other. One explanation for this is that IL‐17 is primarily synthesized in these cells during their activation process in inflamed tissues. The other explanation is the possibility that increased release of IL‐17 from Th17 cells and activated MCs is followed by the uptake of IL‐17 by both cell types. The above‐mentioned study by our group strengthens the idea that Th17 contributes to this cross talk, but more studies are required to establish this assumption [53]. In a recent study, we investigated the possibility that chemokines expressed in lesional CSU skin contributes to T/mast cell proximity. Biopsies from the skin of severe CSU patients were compared to those of healthy individuals. The expression of CCR5 on CD4+ T cells was found to be significantly increased along with its ligand CCL3 on *MCs*. In this respect, CCR5+ T cells were noticed to be in close proximity to CCL3+ *MCs,* suggesting these chemokines play a role in establishing this close contact. Our findings are in full agreement with other reports in which CXCL1/2, CXCL8, and CCR5 are increasingly released from immune cells in both peripheral blood and lesional skin of CSU patients, strengthening the significant role of the crosstalk between immune cells in the pathogenesis of CSU [54]. The involvement of other receptors and or ligands expressed on T cells and *MCs* in CSU should be further assessed to complete the picture of this immune synapse. Of these, the role of the Tec family of tyrosine kinases such as ITK, Tec, Bruton Tyrosin Kinase (Btk) and Bmx are a large group of cytoplasmic protein tyrosin kinases expressed in different immune cell subsets including B cells, T cells, macrophages and MCs*. Btk signaling* in MCs and T cells was shown to take place through different receptors such as FcεRI, T cell receptor (TCR), chemokine receptors and TLRs. These signaling pathways play a role in promoting immune‐mediated inflammation and the crosstalk between immune cells, which infiltrate the skin of CSU patients [55]. The selective blocking of Btk signaling may down regulate the above receptors, thus becoming a possible beneficial therapy for CSU.

## 5 Upcoming Therapeutic Strategies for Targeting Immune Cell Complexity in the Skin of CSU Patients

**FIGURE 2:**
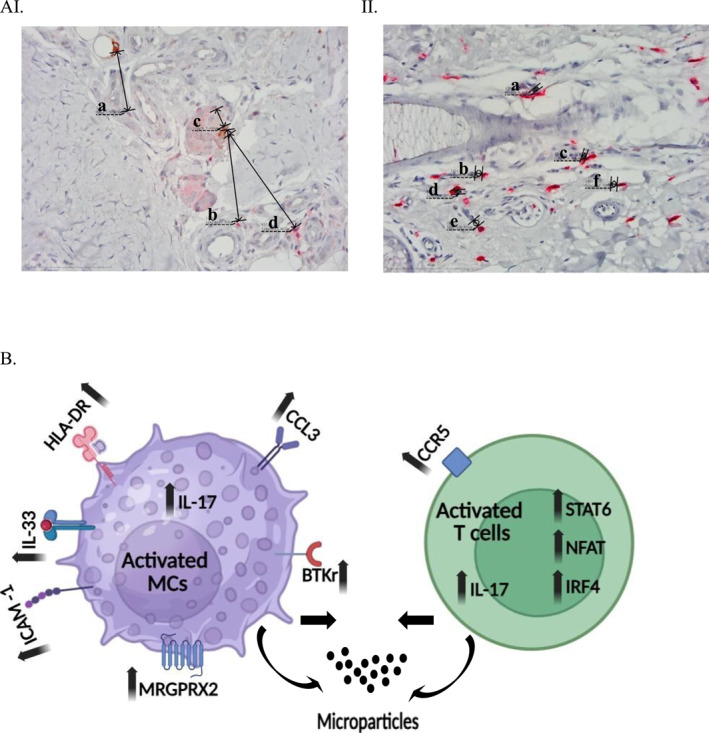
(A) I: Normal skin T/Mast cell proximity measurements: (a) 6.865 µm, (b) 10.518 µm, (c) 2.267 µm, (d) 13.067. II: CSU skin T/Mast cell proximity measurements: (a) 0.465 µm, (b) 0.840 µm, (c) 0.465 µm, (d) 0.507 µm, (e) 0.853 µm, (f) 0.724. × 400 magnification. Scale bar = 40 μm. (B) Immune synapse of activated T and mast cells in the skin of CSU patients.

Following our better understanding of the above described complexity of the crosstalk between all immune cells in lesional CSU skin, many upcoming therapies are currently evaluated to be induced in treating severe CSU, mainly those who are resistant to omalizumab. One of these is anti‐IL‐4 and IL‐13 antibodies (Dupilumab) proved efficient by recent clinical studies, and pointing to the important role of Th2 cells in the pathogenesis of CSU [56]. Increasing evidence supports the notion that Btk inhibitors (Remibrutinib) are highly effective drugs in CSU, acting by various mechanisms such as regulating T‐cell activity, T‐cell repertoire and the inhibition of interleukin‐2 inducible T‐cell kinase (ITK). Btk inhibitors, down regulate inflammatory activities of B cells and innate immune cells such as basophils and MCs. They also inhibit IgE‐mediated FcεR‐dependent immune mechanisms in human MCs [57, 58]. Our report on the beneficial response to anti‐IL‐17 (Secukinumab) in severe CSU patients strengthened the role of IL‐17 in the pathogenesis of CSU, specifically its possible contribution to the close proximity of T/mast cells [53]. The role of JAK‐inhibitors in blocking downstream cytokine signaling pathways, raised their therapeutic potential in targeting T/mast cell interaction in CSU. Abrocitinib and tofacitinib were reported to be beneficial in omalizumab refractory cases of CSU, suggesting that these drugs may become highly relevant in treating severe cases of CSU [59, 60] MCs could also be activated via mechanisms that bypass the high‐affinity IgE receptor and are independent of the autoimmune mechanisms. Among these, we should mention mechanisms that may lead to histamine release such as the coagulation cascade and the activation of the complement system. The role of dedicator of cytokinesis 2 (DOCK2) which is expressed on T cells and its possible effect on the activation of MRGPRX2 which is expressed on MCs is an additional mechanism that contributes to T cell/mast cell proximity. The better definition of extra mechanisms, which lead to T/mast cell proximity, mainly those independent of FcεRI‐mediated ones, should also include GPR15L, which is expressed on dermal T cells, and contributes to the activation of MRGPRX2 receptors expressed on MCs, and their degranulation [61, 62] (Figure 2).

## 6 Conclusion

The cross talk and proximity between T/mast cells in skin inflammation is well established, mainly, the role of microparticles' secretion from both activated T cells and MCs. In this review, we aimed to focus on the importance of this cross talk in lesional skin of CSU patients. The activation of T‐cell subsets and their recruitment to the skin appears to be crucial for this process. The involvement of many cytokines/chemokines such as Th2, Th17 related, but also other molecules such as Btk, JAK‐mediated signaling pathways and MRGPRX2, makes this crosstalk highly complicated. On the other hand, it opens the window for many new upcoming therapies which may become alternative drugs in omalizumab refractory patients.

## Author Contributions

**Elias Toubi:** conceptualization, methodology, supervision, writing – review and editing, writing – original draft, investigation, data curation. **Raeda Mubariki:** investigation, writing – original draft, writing – review and editing. **Zahava Vadasz:** conceptualization, investigation, writing – original draft, writing – review and editing, methodology, validation, formal analysis, supervision, data curation.

## Funding

The authors have nothing to report.

## Conflicts of Interest

The authors declare no conflicts of interest.

## Data Availability

The data that support the findings of this study are available from the corresponding author upon reasonable request.
